# Cardiac Autonomic Response to Active Standing in Calcific Aortic Valve Stenosis

**DOI:** 10.3390/jcm10092004

**Published:** 2021-05-07

**Authors:** José M. Torres-Arellano, Juan C. Echeverría, Nydia Ávila-Vanzzini, Rashidi Springall, Andrea Toledo, Oscar Infante, Rafael Bojalil, Jorge E. Cossío-Aranda, Erika Fajardo, Claudia Lerma

**Affiliations:** 1 Department of Electromechanical Instrumentation, Instituto Nacional de Cardiología Ignacio Chávez, Mexico City 14080, Mexico; jose190288@live.com.mx (J.M.T.-A.); osinfa@yahoo.com (O.I.); 2 Programa de Doctorado en Ciencias Médicas, Odontológicas y de la Salud, Universidad Nacional Autonoma de Mexico, Mexico City 04510, Mexico; 3 Department of Electrical Engineering, Universidad Autónoma Metropolitana, Unidad Iztapalapa, Mexico City 09340, Mexico; 4 Department of Outpatients Clinic, Instituto Nacional de Cardiología Ignacio Chávez, Mexico City 14080, Mexico; vazzny74@yahoo.com (N.Á.-V.); doctorjorgecossio@yahoo.es (J.E.C.-A.); fajardoerikaf@yahoo.com (E.F.); 5 Department of Immunology, Instituto Nacional de Cardiología Ignacio Chávez, Mexico City 14080, Mexico; raspringall@yahoo.com (R.S.); andtole@gmail.com (A.T.); 6 Department of Health Care, Universidad Autónoma Metropolitana, Unidad Xochimilco, Mexico City 04960, Mexico; rafaelbojalil@gmail.com

**Keywords:** aortic valve disease, cardiac autonomic modulation, active standing

## Abstract

Aortic stenosis is a progressive heart valve disorder characterized by calcification of the leaflets. Heart rate variability (HRV) analysis has been proposed for assessing the heart response to autonomic activity, which is documented to be altered in different cardiac diseases. The objective of the study was to evaluate changes of HRV in patients with aortic stenosis by an active standing challenge. Twenty-two volunteers without alterations in the aortic valve (NAV) and twenty-five patients diagnosed with moderate and severe calcific aortic valve stenosis (AVS) participated in this cross-sectional study. Ten minute electrocardiograms were performed in a supine position and in active standing positions afterwards, to obtain temporal, spectral, and scaling HRV indices: mean value of all NN intervals (meanNN), low-frequency (LF) and high-frequency (HF) bands spectral power, and the short-term scaling indices (α_1_ and α_sign1_). The AVS group showed higher values of LF, LF/HF and α_sign1_ compared with the NAV group at supine position. These patients also expressed smaller changes in meanNN, LF, HF, LF/HF, α_1,_ and α_sign1_ between positions. In conclusion, we confirmed from short-term recordings that patients with moderate and severe calcific AVS have a decreased cardiac parasympathetic supine response and that the dynamic of heart rate fluctuations is modified compared to NAV subjects, but we also evidenced that they manifest reduced autonomic adjustments caused by the active standing challenge.

## 1. Introduction

Calcific aortic valve disease is a progressive asymptomatic condition that begins with inflammation and lipid infiltration of the aortic valve leaflets as well as focal or diffuse calcium accumulation, which eventually leads to stenosis of the aortic valve in 1.8% to 1.9% of patients per year [1,2]. In many patients, the diagnosis of aortic valve stenosis (AVS) is secondary to the first signs or symptoms during the advanced stages of the disease, when mortality risk is very high if an aortic valve replacement surgery is not carried out [3]. Although the prevalence of AVS increases with age, other associated factors include hypertension, dyslipidemia, smoking, and diabetes mellitus [4,5,6].

The modulation exerted by the autonomic nervous system is considered to have a crucial role in the adequate response of blood vessels and heart activity to both daily and unexpected challenges [7]. Notwithstanding that autonomic modulation provides the cardiovascular system with significant adaptive responses, its impairment could also be involved in the etiology or progression of several cardiovascular diseases [8,9]. For instance, in essential hypertension, predominance of the sympathetic nervous system seems to initiate or sustain the arterial stiffness and rigidity, combined with higher inotropic and chronotropic activity of the heart [10].

There are few studies regarding the autonomic modulation in patients with aortic valve disease, most of them based on heart rate variability (HRV) analysis of ambulatory long-term recordings from patients with AVS [11,12,13,14,15]. However, such recordings were obtained with no control (or at least registration) of patients’ activity, which weakens the interpretation of the HRV analysis in terms of the cardiac autonomic modulation [9]. Yet, all these studies agree with the observation of an altered HRV (based on different indices), which is considered to provide evidence of the increased sympathetic nervous activity in conjunction with decreased vagal activity of the heart in AVS [10].

The orthostatic challenge is a physiological stimulus that can be used to assess autonomic cardiac modulation by comparing the HRV indices in a baseline condition (i.e., supine position) with the resulting indices after experiencing the hemodynamic stimulus (i.e., the upright position) [16,17,18]. In healthy subjects, the heart rate modulation during supine position is characterized by an important parasympathetic (vagal) response [19,20]. In response to the orthostatic stimulus, the vagal response decreases and the sympathetic one becomes more important, which is manifested by a HRV reduction showing a predominance of low-frequency over high-frequency oscillations [21,22]. This HRV reduction caused by the orthostatic challenge also introduces a distinctive dynamical change in such oscillations [23,24,25]. To characterize this behavior, the so-called scaling indices can be used [26,27], which quantify the fractal-like HRV irregularity along different time scales.

Patients with aortic valve sclerosis (an early stage of AVS) show a predominance of low-frequency oscillations in a supine position, and smoother and less anti-correlated behavior as shown by the scaling indices, all indicating an increased sympathetic cardiac autonomic modulation. They also present smaller changes of HRV indices in response to active standing (compared to healthy subjects), perhaps suggesting a decreased adjustment to this hemodynamic challenge [28]. Yet, the autonomic cardiac response to active standing has not been studied in patients with calcific AVS. Therefore, the aim of this work was to evaluate the cardiac autonomic response during an active standing challenge in these patients, in comparison with subjects with a healthy aortic valve as revealed by 2D transthoracic echocardiograms.

## 2. Materials and Methods

### 2.1. Subjects and Study Protocol

A cross-sectional study was carried out at the National Cardiology Institute “Ignacio Chávez” with healthy volunteers and aortic valve stenosis patients, who had an age range of 30 to 80 years. Exclusion criteria were any ischemic, renal, inflammatory, or autoimmune diseases, or moderate or significant injury in the mitral or tricuspid valves. None of these participants received β-blockers medication. Volunteers who were considered healthy were recruited after an invitation to the institute alongside staff and patient’s relatives. They had no known comorbidities and were not taking any drugs. In a total of 98 volunteers, the absence of comorbidities was confirmed by a clinical screening. Afterwards, an echocardiogram was performed to each volunteer and 76 participants were excluded due to the presence of any aortic valve sclerosis (either grade 1 or 2 [29]) as revealed by calcified focal areas of increased echogenicity and thickened aortic-valve leaflets [30,31]. At the end, we obtained a group of 22 healthy volunteers with no abnormalities in the aortic valve, i.e., the normal aortic valve (NAV) group. A total of 39 patients previously diagnosed with aortic valve stenosis were invited to participate in the study during a follow-up visit to the outpatients clinic of the institute, being candidates for the elective valve replacement program. Eight were excluded due to the finding of other valvular heart diseases during echocardiography. Five more, who had diagnosed diabetes mellitus, were eliminated from the study, and we ended up with 25 patients in the aortic valve stenosis (AVS) group. Our study was performed before aortic valve replacement for those who enter such program. Previous history of hypertension, dyslipidemia, alcoholism, and smoking was obtained from clinical records.

Anthropometric measures, oscillometric blood pressure, and a resting electrocardiogram (ECG) of 12 leads were obtained. A second continuous ECG recording was performed with a chest band (BioHarness 3.0, Zephyr Technology, Annapolis, MD, USA) while participants remained in a supine position for 10 min, followed by active standing for another 10 min. Whilst in a supine position, participants were asked to lie with legs uncrossed and hands by their sides. Finally, a 2D transthoracic echocardiogram was performed.

### 2.2. Echocardiographic Assessment and Study Groups

One specialist measured the echocardiographic parameters by two-dimensional Doppler, employing a commercial machine (iE33, Philips Healthcare, Bothell, WA, USA). Using a pulsed wave Doppler recording, the following echocardiographic parameters were obtained: maximum aortic valve transvalvular velocity in meters per second and mean and maximum mean pressure gradient (mmHg), aortic valve area (cm^2^), and left ventricular ejection fraction (%).

### 2.3. Electrocardiogram Recording and HRV Indices

From continuous ECG recording, the QRS complex for each heartbeat was identified with our custom-made computer program previously validated [32]. Then, artifacts and ectopic beats were identified visually to eliminate artifacts and RR intervals derived from ectopic beats and to obtain the HRV time series only from complexes of a sinus node origin (NN intervals) [33]. Time series of 300 NN intervals were selected from both positions (supine position and active standing) choosing stable segments after the first 180 s in each position. All series were fixed to this number of intervals to cover ~5 min and to avoid introducing variations in the estimation of the scaling indices, described below, which would result from analyzing series having a different number of NN intervals [34].

**Figure 1:**
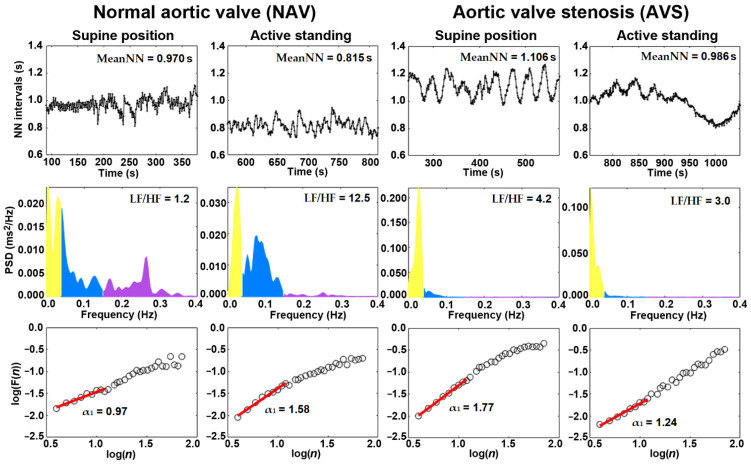
Example of time series (upper panel), power spectrum density (middle panel) and detrended fluctuation analysis (DFA) plot (low panel) from one participant with normal aortic valve (NAV) and one patient with aortic stenosis (AVS). MeanNN: mean value of all NN intervals; PSD: power spectral density; LF/HF: ratio between low-frequency (LF) band and high-frequency (HF) band indices; α_1_: short-term scaling index (from the DFA plot).

For each NN time series, the HRV analysis was applied following the international recommendations [33] and in accordance with previous studies [24,28]. The following time-domain HRV indices were calculated: meanNN (mean value of all NN intervals), SDNN (standard deviation of all NN intervals), root mean squared of the successive differences (RMSSD), and pNN20 (percentage of successive NN intervals with differences greater than 20 ms). Figure 1 shows an example of the HRV time series from one healthy subject and one patient with AVS (upper panel). For the estimation of the frequency domain indices, each time series was resampled with a line interpolation method at 3 samples per second and the power spectrum density was obtained using Welch’s periodogram (Figure 1, middle panel). The mean spectral power was obtained for the low frequency band (LF, 0.04 to 0.15 Hz), which has been associated with both sympathetic and parasympathetic activity, and for the high-frequency band (HF, 0.15 to 0.4 Hz), which is considered a reliable parameter related to vagal activity [10]. LF and HF were transformed into normalized units (nu) [33]. The scaling indices α_1_ and α_1sign_ were calculated for each original HRV time series with detrended fluctuation analysis (DFA) within a short range of time scales covered by 4 to 11 NN intervals [26]. The scaling indices α_1_ and α_1sign_ quantify fractal-like the irregularity that occurs in HRV through different time scales, which are related to the presence or absence of scaling correlation properties (α_1_) and the directionality (α_1sign_) of the HRV time series [23,26,27]. These scaling indices are considered to be reliable dynamical features to characterize HRV time series [35,36] and show a consistent correlation or covariance with the mean heart rate, both in healthy subjects and end-stage renal disease patients [24].

For all HRV indices obtained here, we calculated a magnitude of change (Δ) resulting from the difference between the values in the supine position and the values during active standing. The HVR indices estimation was performed with ad hoc computer programs developed in Matlab version R2018a (MathWorks, Inc., Natick, MA, USA).

### 2.4. Statistical Analysis

For continuous variables, a Kolmogorov–Smirnov test was applied to determine if they had a normal distribution. For variables with normal distribution, the results are reported as mean ± standard deviation, and were compared between groups using Student’s *t*-test or analysis of variance for repeated measures with one factor of comparison between subjects (NAV or AVS group) and one factor of comparison within subjects (supine position and active standing). The variables that had no normal distribution are reported as median (percentile 25–percentile 75) and were compared between groups by the Mann–Whitney U test, Wilcoxon Rank’s test or Kruskal–Wallis test. Nominal variables are reported by absolute values (percentage) and were compared between groups by Chi-squared test. Multiple linear stepwise regression models without interactions were performed to evaluate whether there was a relationship between the changes (Δ) of each HRV index in response to active standing (as dependent variable) with the change of meanNN, age, systolic blood pressure and the condition of stenosis (as independent variables). Additional models were also analyzed in which systolic blood pressure was substituted with statins or aspirin use. Finally, linear regression analysis for all HRV indices were also tested with ΔmeanNN and the propensity score as independent variables. This propensity score was computed using binary logistic regression analysis, as the conditional probability of the AVS presence, given the following covariates: age, systolic blood pressure, statins use, aspirin use, and serum glucose [37]. The statistical analysis was performed by SPSS version 21.0 (IBM Corp., Armonk, NY, USA), and a value of *p <* 0.05 was considered as statistically significant.

## 3. Results

Table 1 shows the sociodemographic characteristics and risk factors of the study participants. Compared with the subjects with a normal aortic valve, the patients with aortic valve stenosis were older, had higher systolic blood pressure, and more cases with hypertension, dyslipidemia, and prescribed drug intake (statins and aspirin). There were no significant differences in the other variables.

The echocardiographic parameters are consistent with the selection criteria of the study: compared to the normal aortic valve group, the patients with aortic valve stenosis had smaller AVA, AVAi and LVEF, as well as larger Vmax, AVG mean, AVGmax (Table 2). According to these parameters, our AVS patients were considered to manifest a moderate (*n* = 11) and severe condition (*n* = 14) [38].

From the laboratory results, we observed that the aortic valve stenosis group had higher serum glucose levels compared to the normal aortic valve group, while for the rest of the characteristics there were no significant differences (Table 3).

Table 4 shows the results of the cardiac autonomic activity evaluation through the HRV indices during the orthostatic challenge. The p-values correspond to the comparisons between groups (within the same position). Within the supine position, patients of the AVS group showed smaller RMSSD and HF, as well as larger LF, LF/HF, and α_1sign_, compared to the subjects with normal aortic valves. All other HRV indices were similar between groups whilst in a supine position. After active standing, patients with aortic valve stenosis had larger RMSSD, α_1_ and α_1sign_. All other HRV indices were similar between groups after active standing. The comparisons of the response to the orthostatic challenge (within each group) showed significant changes in most HRV indices (indicated by asterisks). The NAV subjects had a decrement in meanNN, pNN20, RMSSD and HF, as well as an increment in LF, LF/HF, α_1_ and α_1sign_. Only the SDNN index did not change in response to active standing in the NAV group. AVS patients had decrement in meanNN, SDNN, pNN20, RMSSD and HF, as well as increment in LF. These patients had no significant change in the other HRV indices (LF/HF, α_1_ and α_1sign_) in response to active standing.

Compared with the NAV group, the AVS group had significantly lower changes of Δ (the difference between the values in the supine position and the values after active standing) in response to active standing for all HRV indices (ΔmeanNN, ΔLFnu, ΔHFnu, ΔLF/HF, Δα_1_ and Δα_1sign_) except ΔSDNN, ΔpNN2O and ΔRMSSD (Table 5).

According to the multiple linear regression analysis (Table 6), the change of meanNN (ΔmeanNN) was a factor associated with the changes in pNN20 (ΔpNN20) and RMSSD (ΔRMSSD), while ΔmeanNN and having the condition of calcific aortic valve stenosis were independent factors associated with the active standing changes in LF (ΔLF), HF (ΔHF) and α_1_ (Δα_1_).

Additional linear stepwise multiple regression analyses were performed to assess either statins or aspirin use (as dichotomized variables), as is shown in the supplementary material. For all HRV indices, as dependent variables, similar models were obtained, and the statins use (Table S1) or aspirin use (Table S2) did not contribute to the dependent variables. Furthermore, the linear stepwise multiple regression analyses with models that consider both the ΔmeanNN and the propensity score as independent variables also showed similar results for all HRV indices (Table S3).

The comparisons of the study variables between patients with moderate AVS and severe AVS are also shown in the supplementary material. Compared to moderate AVS patients, those with severe AVS had similar characteristics and risk factors (Table S4), but higher atherogenic index (Table S5), larger LF and LF/HF (during supine position), and larger RMSSD (during active standing) (Table S6). All other variables were similar between patients with moderate and severe AVS, including the magnitude of change (Δ) of all HRV indices (Table S7).

## 4. Discussion

By evaluating the heart rate variability in patients with calcified aortic valve stenosis (AVS), in short recordings and during controlled activity conditions, we confirmed that patients at supine position show a less predominant cardiac response to the parasympathetic modulation in comparison with a healthy valve group (NAV). This diminished predominance was manifested without involving differences in the mean heart rate at supine position and coincides with previous studies of ambulatory long records in AVS patients [11,12,13,14,15]. We also identified a dynamic behavior of HRV that reflects less anti-correlation (i.e., greater α_1sign_) in AVS at the supine position. In addition, as a novel finding we evidenced that AVS patients show reduced changes in HRV indices by the active standing challenge.

The active standing maneuver produced a heart rate increase in both groups and a higher predominance of the cardiac response to the sympathetic modulation, but only in the NAV group significant changes were observed in the scaling indices related to the dynamic behavior of heart rate fluctuations, with increments of α_1_ and α_1sign_. Interestingly, after standing, both groups achieved a similar sympathetic predominance according to the LF/HF index. However, when comparing the magnitude of the differences (Δ) in the HRV indices between positions, the response to standing in the cardiac autonomic regulation was clearly lower in patients with stenosis, with significant differences of this magnitude in meanNN, LF, HF, LF/HF, α_1_ and α_sign1_ (Table 5). This suggests that confronted with the orthostatic challenge, patients show restricted autonomic adjustments. The reason for these lower adjustments could be identified through modified [14] autonomic activity resulting in left ventricular remodeling and hypertrophy of the AVS patients [39], but also in other adaptations that could be needed to avoid increasing blood pressure once standing. Our AVS group indeed presented significantly higher systolic blood pressure (SBP) values, and greater cases with diagnosed hypertension (Table 1), which is identified as a concomitant manifestation of AVS [6]. Some studies of patients with essential hypertension have documented that this last condition is associated with smaller changes of the meanNN provoked by an orthostatic challenge [40,41]. However, according to our multiple linear regression results, neither the age nor the SBP differences between groups were associated with the HRV adjustments caused by the active standing challenge (Table 6).

In earlier studies of AVS patients, authors have also reported differences in HRV indices. By analyzing 2 h segments, Vukasovic et al. found increased power of the LF frequency band in severe AVS patients in comparison with a control group (7.5 ± 1.8 ms vs. 4.9 ± 1.7 ms). However, eight to ten months after valve replacement, an increase in “heart rate variability” was observed (from 50 ± 22 ms to 79.5 ± 22 ms) [11]. Similar findings were reported by Arslan et al. [12], who documented, from 43 mild and moderate AVS patients and 50 controls, differences in the LF and LF/HF indices (27.5 ± 7.9 vs. 20.7 ± 4.7 nu; 3.7 ± 1.3 vs. 2.0 ± 0.7, respectively) as well as in HF (8.8 ± 2.2 vs. 13.1 ± 3.1 nu). Zuern et al. [14] found in patients with moderate and severe AVS, diagnosed with dysautonomia (severe autonomic failure), that 42% of cases presented lower HRV and 48.1% lower LVEF. Finally, Werner et al. [13] analyzed HRV data collected from children with aortic valve stenosis and found diminished indices associated with parasympathetic activity. In children with AVS, short-term recordings during tilting tests have shown that passive standing increases LF and decreases HF during the maneuver’s phase two (i.e., after 10 min of tilt), while children from a control group manifested these changes earlier [42]. The late response in AVS children was interpreted as a delayed cardiac response to sympathetic modulation. In our study with adult AVS patients, the active standing test lasted a total of 10 min and the HRV indices were measured during the second half of the standing position. Therefore, it is likely that the attenuated responses in HRV of our patients reported here also occurred during the equivalent phase two of the active orthostatic challenge. However, we did not perform analysis on earlier stages of the standing test, and further research is necessary to conclude that such attenuated responses to active standing were also delayed compared to NAV subjects.

The scaling indices α_1_ and α_1sign_ used here have not been reported in previous studies analyzing data from calcific AVS patients. Yet, both allowed us to identify that the dynamic behavior of heart rate fluctuations in these patients is also modified. NAV subjects showed larger anti-correlated behavior at supine position (see α_1sign_ in Table 4) and only for these people did we find significant increments in both α_1_ and α_1sign_ (i.e., larger regularity and less anti-correlation) as would be expected by the active standing maneuver [24,25]. An anti-correlated behavior of heart rate fluctuations can be attributed to a condition in which a modulating factor (e.g., the parasympathetic activity) exerts a dominant effect, whereas less anti-correlation is manifested when various regulatory factors participate [43,44]. Formerly, we have reported differences in α_1_ and α_1sign_ at supine position as studied in subjects already showing aortic valve sclerosis but without any clinical manifestation of stenosis, which also indicated the manifestation of a smoother and less anti-correlated HRV behavior in this precursory condition of aortic valve disease [28]. Some authors have identified the scaling indices as independent predictors of mortality in relation to sudden cardiac death, chronic heart failure, dilated cardiomyopathy and end-stage renal disease [35,36,45].

### Study Limitations

Changes in HRV indices have been identified in several other conditions such as diabetes, hypertension, dyslipidemia or obesity [7,46,47]. Given that these conditions are recognized as some of the concomitant risk factors for the manifestation and progression of valvular calcification [46,48,49], further research enrolling a larger number of cases is required to elucidate how such factors could be involved in the differences and attenuated response to active standing reported here. Other important factors to consider and their interactions are age, male gender, smoking or any pharmacological treatment including statins and aspirin, associated with the common comorbidities in AVS patients [50]. However, our multiple regression models do suggest that neither age, statins, aspirin nor SBP differences were associated with the reduced adjustments in AVS patients that we are reporting. We excluded from our study AVS patients medicated with β-blockers as well.

## 5. Conclusions

In conclusion, we confirmed in short-term recordings that patients with AVS have a decreased cardiac parasympathetic supine response compared to NAV subjects, but we also evidenced that they manifest reduced HRV adjustments caused by the active standing challenge. Given that these reduced adjustments were not associated with the age or SBP differences between groups, a modified autonomic activity seems to be involved in patients with moderate and severe calcific AVS.

## Figures and Tables

**Table 1:** Characteristics and risk factors of participants. Data are shown as absolute value (percentage), mean ± standard deviation, or median (percentile 25–percentile 75).

Variable	NAV (*n* = 22)	AVS (*n* = 25)	*p* Value
Age (years)	41 ± 8	63 ± 7	<0.001
Female Male	10 (45%) 12 (55%)	8 (32%) 17 (68%)	0.259
Body mass index (kg/m^2^)	27.35 ± 3.69	28.34 ± 3.56	0.354
Heart rate (bpm)	60.8 ± 9.7	62.2 ± 11.3	0.654
SBP (mmHg)	112 ± 11	136 ± 20	<0.001
DBP (mmHg)	78 (70–80)	80 (76–90)	0.083
Hypertension	2 (9%)	11 (44%)	0.008
Dyslipidemia	0 (%)	7 (28%)	0.008
Alcoholism	10 (46%)	13 (52%)	0.438
Smoking	6 (32%)	8 (32%)	0.488
Statins	0 (0%)	5 (20%)	0.035
Aspirin	0 (0%)	10 (40%)	0.001

NAV: normal aortic valve; AVS: aortic valve stenosis; SBP: systolic blood pressure; DBP: diastolic blood pressure.

**Table 2:** Parameters evaluated from the echocardiogram. Data are shown as median (percentile 25–percentile 75).

Variable	(NAV) (*n* = 22)	(AVS) (*n* = 25)	*p* Value
AVA (cm^2^)	4.20 (4.03–4.20)	0.60 (0.41–1.21)	<0.001
AVAi (cm^2^/m^2^)	2.17 (2.06–2.38)	0.36 (0.25–0.71)	<0.001
Vmax (m/s)	1.20 (1.02–1.37)	4.30 (3.18–5.37)	<0.001
AVGmean (mmHg)	3 (2–3)	43 (23–70)	<0.001
AVGmax (mmHg)	5 (4–7)	74 (38–115)	<0.001
LVEF (%)	62 ± 6	54 ± 9	<0.001

NAV: normal aortic valve; AVS: aortic valve stenosis; AVA = aortic valve area; AVAi: indexed aortic valve area; Vmax: aortic-valve maximum flow velocity; AVGmean: aortic-valve mean gradient; AVGmax: maximum gradient of the aortic valve; LVEF: left ventricular ejection fraction.

**Table 3:** Biochemical parameters of the study participants. Data are shown as mean ± standard deviation or median (percentile 25–percentile 75).

Variable	NAV (*n* = 22)	AVS (*n* = 25)	*p* Value
Serum glucose (mg/dL)	87.7 ± 12.1	97.6 ± 11.3	<0.008
Albumin (mg/dL)	4.39 ± 0.22	4.48 ± 0.32	0.413
Total cholesterol (mg/dL)	191.76 ± 34.25	182.08 ± 37.37	0.359
High density lipids (mg/dL)	41.59 ± 10.54	42.82 ± 11.69	0.706
Low density lipids (mg/dL)	125 ± 32	107 ± 36	0.065
Triglycerides (mg/dL)	139 (113–163)	151 (106–189)	0.579
Atherogenic index	3.21 ± 1.19	2.72 ± 1.35	0.193
C-reactive protein (mg/dL)	2.60 (1.30–3.4)	2.00 (0.89–4.17)	0.880
Hemoglobin (mg/dL)	15.0 ± 1.7	14.7 ± 1.5	0.586
Hematocrit (%)	45.1 ± 4.3	43.6 ± 4.6	0.271

NAV: normal aortic valve; AVS: aortic valve stenosis.

**Table 4:** Heart rate variability indices at supine position and after active standing. Data are shown as mean ± standard deviation, or median (percentile 25–percentile 75). The groups were compared with analysis of variance for repeated measures or Kruskal–Wallis test, Mann–Whitney U test and Wilcoxon Rank’s test.

Variable	NAV (*n* = 22)	AVS (*n* = 25)	*p* Value
Supine position			
MeanNN (s)	0.994 ± 0.180 **	0.987 ± 0.167 **	0.890
SDNN (ms)	54.3 ± 23.5	50.3 ± 26.3 *	0.591
pNN20 (%)	59.0 ± 31.2 **	41.2 ± 34.5 **	0.058
RMSSD (ms)	38.7 ± 16.9 **	26.3 ± 13.3 **	0.007
LF (nu)	56.8 (44.0–68.5) **	76.7 (54.2–84.7) *	0.004
HF (nu)	43.2 (31.5–56.0) **	23.3 (15.3–45.8) *	0.004
LF/HF	1.31 (0.78–2.17) **	3.29 (1.18–5.53)	0.004
α_1_	0.993 ± 0.21 **	1.16 ± 0.42	0.083
α_1sign_	0.171 ± 0.14 **	0.33 ± 0.23	0.006
Active standing			
MeanNN (s)	0.825 ± 0.15	0.888 ± 0.12	0.132
SDNN (ms)	53.3 ± 30.6	41.8 ± 18.4	0.138
pNN20 (%)	40.5 ± 26.9	28.9 ± 20.4	0.108
RMSSD (ms)	26.3 ± 13.9	19.1 ± 9.3	0.048
LF (nu)	85.4 (73.2–88.6)	82.2 (70.8–87.4)	0.277
HF (nu)	14.6 (11.4–26.8)	17.8 (12.6–29.2)	0.277
LF/HF	5.83 (2.73–7.74)	4.60 (2.42–6.92)	0.277
α_1_	1.40 ± 0.21	1.24 ± 0.32	0.038
α_1sign_	0.466 ± 0.14	0.366 ± 0.13	0.019

NAV: normal aortic valve; AVS: aortic valve stenosis; meanNN; mean value of all NN intervals; SDNN: standard deviation of all NN intervals; RMSSD: root mean squared of the successive differences; pNN20: percentage of successive NN intervals with differences greater than 20 ms), LF: low-frequency band spectral power; HF: high-frequency band spectral power; nu: normalized units; LF/HF: ratio between low-frequency and high frequency band indices; α_1_: short-term scaling index; and α_1sign_: short-term scaling index from the sign time series. * *p <* 0.05 compared to active standing (within same group); ** *p* < 0.01 compared to active standing (within same group).

**Table 5:** Magnitude (Δ) of change in heart rate variability indices in response to active standing. Data are shown as mean ± standard deviation and were compared between groups by a student *t*-test for independent groups.

Variable	NAV (*n* = 22)	AVS (*n* = 25)	*p* Value
ΔmeanNN (s)	0.170 ± 0.070	0.100 ± 0.100	0.010
ΔSDNN (ms)	1 ± 18	8 ± 23	0.221
ΔpNN20 (%)	18.53 ± 12.55	12.28 ± 23.17	0.250
ΔRMSSD (ms)	12.48 ± 10.06	7.24 ± 11.47	0.102
ΔLF (nu)	−26.48 ± 18.03	−7.03 ± 15.92	<0.001
ΔHF (nu)	26.52 ± 18.06	7.05 ± 15.91	<0.001
Δ(LF/HF)	−5.20 ± 4.52	−0.937 ± 3.77	<0.001
Δα_1_	−0.42 ± 0.23	−0.07 ± 0.32	<0.001
Δα_1sign_	−0.29 ± 0.20	−0.03 ± 0.22	<0.001

NAV: normal aortic valve; AVS: aortic valve stenosis. Δ: difference between the values in the supine position and the values after active standing in each HRV index; ΔmeanNN: change of meanNN; ΔSDNN: change of SDNN; ΔRMSSD: change of RMSSD; ΔpNN20: change of pNN20, ΔLF: change of LF; ΔHF: change of HF; nu: normalized units; Δ(LF/HF): change of LF/HF ratio; Δα_1_: change of α_1_; and Δα_1sign_: change of α_1sign_.

**Table 6:** Linear stepwise multiple regression analysis with predicted heart rate variability (HRV) indices, and independent variables: ΔmeanNN (s), the aortic valve stenosis (AVS) condition (dichotomized), systolic blood pressure (SBP, mmHg) and age (years).

Variables	Standardized β	β (C.I._95%_)	*p*	R^2^
Predicted HRV index: ΔpNN20				0.409
ΔmeanNN	0.650	126.67 (79.98–173.37)	<0.001	
AVS condition	Excluded variable			
Age	Excluded variable			
SBP	Excluded variable			
Predicted HRV index: ΔRMSSD				0.249
ΔmeanNN	0.516	57.02 (27.19–86.85)	<0.001	
AVS condition	Excluded variable			
Age	Excluded variable			
SBP	Excluded variable			
Predicted HRV index: ΔLF				0.365
ΔmeanNN	−0.353	−65.53 (−114.50–−16.55)	0.010	
AVS condition	0.415	7.54 (2.74–12.33)	0.003	
Age	Excluded variable			
SBP	Excluded variable			
Predicted HRV index: ΔHF				0.367
ΔmeanNN	0.355	66.12 (17.11–115.1)	0.009	
AVS condition	−0.414	−7.54 (−12.34–−2.74)	0.003	
Age	Excluded variable			
SBP	Excluded variable			
Predicted HRV index: ΔLF/HF				0.202
ΔmeanNN	Excluded variable			
AVS condition	0.471	2.16 (0.88–3.44)	0.001	
Age	Excluded variable			
SBP	Excluded variable			
Predicted HRV index: Δα_1_				0.437
ΔmeanNN	−0.447	−1.51 (−2.35–−0.67)	0.001	
AVS condition	0.385	0.12 (0.04–0.21)	0.003	
Age	Excluded variable			
SBP	Excluded variable			
Predicted HRV index: Δα_1sign_				0.331
ΔmeanNN	Excluded variable			
AVS condition	0.589	0.141 (0.08–0.20)	<0.001	
Age	Excluded variable			
SBP	Excluded variable			

meanNN; mean value of all NN intervals; RMSSD: root mean squared of the successive differences; pNN20: percentage of successive NN intervals with differences greater than 20 ms), LF: low-frequency band; HF: high-frequency band; nu: normalized units; LF/HF: ratio between low-frequency and high frequency band indices; α_1_: short-term scaling index; and α_1sign_: short-term scaling index from the sign time series.

## Data Availability

The data presented in this study are available on request from the corresponding author.

## Supplementary Materials

The following are available online at https://www.mdpi.com/article/10.3390/jcm10092004/s1, Table S1: Linear stepwise multiple regression analysis with predicted HRV indices, and as independent variables, ΔmeanNN (s), the aortic valve stenosis condition (dichotomized), statins and age (years), Table S2: Linear stepwise multiple regression analysis with predicted HRV indices, and as independent variables, ΔmeanNN (s), the aortic valve stenosis condition (dichotomized), aspirin and age (years), Table S3: Linear stepwise multiple regression analysis with predicted HRV indices, and as independent variables, ΔmeanNN (s) and propensity score, Table S4: Characteristics and risk factors of aortic valve stenosis patients, Table S5: Biochemical parameters of the aortic valve stenosis patients, Table S6: Heart rate variability indices at supine position and after active standing of the aortic valve stenosis patients, Table S7: Magnitude (Δ) of change in heart rate variability indices in response to active standing of the aortic valve stenosis patients.
