# Supplementary material for: Cardiac Autonomic Response to Active Standing in Calcific Aortic Valve Stenosis
Source: J Clin Med. 2021 May 7;10(9):2004. doi: 10.3390/jcm10092004 (PMC8124878; doi:10.3390/jcm10092004)
Supplement: Supplementary file 1 [file jcm-10-02004-s001.zip › jcm-1179906-supplementary.pdf]

Supplementary material

Table S1. Linear stepwise multiple regression analysis with predicted HRV indices, and as independent variables, ΔmeanNN (s), the aortic valve stenosis (AVS) condition (dichotomized), statins and age (years).

Variables	Standardized β	β (C.I.$_{95\%}$)	P	R^2
**Predicted HRV *index:* ΔpNN20**				0.433
ΔmeanNN	0.667	128.57 (85.47 – 171.68)	< 0.001	
AVS condition		*Excluded variable*		
Age		*Excluded variable*		
Statins		*Excluded variable*		
**Predicted HRV *index:* ΔRMSSD**				0.277
ΔmeanNN	0.541	60.49 (32.25 – 88.73)	< 0.001	
AVS condition		*Excluded variable*		
Age		*Excluded variable*		
Statins		*Excluded variable*		
**Predicted HRV *index*: ΔLFnu**				0.344
ΔmeanNN	-0.368	-72.39 (-123.18 - -21.61)	0.006	
AVS condition	0.372	7.15 (2.18 – 12.12)	0.006	
Age		*Excluded variable*		
Statins		*Excluded variable*		
**Predicted HRV *index:* ΔHFnu**				0.346
ΔmeanNN	0.370	72.89 (22.13 – 123.6)	0.006	
AVS condition	-0.371	-7.14 (-12.11 – -2.18)	0.006	
Age		*Excluded variable*		
Statins		*Excluded variable*		
**Predicted HRV *index*: ΔLF/HF**				0.199
ΔmeanNN		*Excluded variable*		
AVS condition	0.465	2.13 (0.91 – 3.35)	0.001	
Age		*Excluded variable*		
Statins		*Excluded variable*		
**Predicted HRV *index:* Δα_1**				0.421
ΔmeanNN	-0.439	-1.45 (-2.25 – -0.65)	0.001	
AVS condition	0.369	0.120 (0.04 – 0.19)	0.004	
Age		*Excluded variable*		
Statins		*Excluded variable*		
Predicted HRV index: Δα_{1sign}				0.270
ΔmeanNN		*Excluded variable*		
AVS condition	0.535	0.131 (0.06 – 0.19)	<0.001	
Age		*Excluded variable*		
Statins		*Excluded variable*		

Table S2. Linear stepwise multiple regression analysis with predicted HRV indices, and as independent variables, ΔmeanNN (s), the aortic valve stenosis (AVS) condition (dichotomized), aspirin and age (years).

Variables	Standardized β	β ($C.I._{95\%}$)	P	R^2
**Predicted HRV *index:* ΔpNN20**				0.433
ΔmeanNN	0.667	128.57 (85.47 – 171.68)	< 0.001	
AVS condition		*Excluded variable*		
Age		*Excluded variable*		
Aspirin		*Excluded variable*		
**Predicted HRV *index:* ΔRMSSD**				0.277
ΔmeanNN	0.541	60.49 (32.25 – 88.73)	< 0.001	
AVS condition		*Excluded variable*		
Age		*Excluded variable*		
Aspirin		*Excluded variable*		
**Predicted HRV *index:* ΔLFnu**				0.344
ΔmeanNN	-0.368	-72.39 (-123.18 - -21.61)	0.006	
AVS condition	0.372	7.15 (2.18 – 12.12)	0.006	
Age		*Excluded variable*		
Aspirin		*Excluded variable*		
**Predicted HRV *index:* ΔHFnu**				0.346
ΔmeanNN	0.370	72.89 (22.13 – 123.6)	0.006	
AVS condition	-0.371	-7.14 (-12.11 – -2.18)	0.006	
Age		*Excluded variable*		
Aspirin		*Excluded variable*		
**Predicted HRV *index:* ΔLF/HF**				0.199
ΔmeanNN		*Excluded variable*		
AVS condition	0.465	2.13 (0.91 – 3.35)	0.001	
Age		*Excluded variable*		
Aspirin		*Excluded variable*		
**Predicted HRV *index:* $\Delta\alpha_1$**				0.421
ΔmeanNN	-0.439	-1.45 (-2.25 – -0.65)	0.001	
AVS condition	0.369	0.120 (0.04 – 0.19)	0.004	
Age		*Excluded variable*		
Aspirin		*Excluded variable*		
**Predicted HRV *index:* $\Delta\alpha_{1sign}$**				0.270
ΔmeanNN		*Excluded variable*		
AVS condition	0.535	0.131 (0.06 – 0.19)	<0.001	
Age		*Excluded variable*		
Aspirin		*Excluded variable*		

Table S3. Linear stepwise multiple regression analysis with predicted HRV indices, and as independent variables, ΔmeanNN (s) and propensity score.

Variables	Standardized β	β (C.I.$_{95\%}$)	P	R^2
**Predicted HRV *index:* ΔpNN20**				0.410
ΔmeanNN	0.651	128.80 (80.21 – 177.39)	< 0.001	
Propensity score		*Excluded variable*		
**Predicted HRV *index:* ΔRMSSD**				0.352
ΔmeanNN	0.593	67.77 (37.40 – 96.14)	< 0.001	
Propensity score				
**Predicted HRV *index:* ΔLFnu**				0.335
ΔmeanNN	-0.370	-70.43 (-144.40 – -16.45)	0.012	
Propensity score	0.339	13.52 (2.21 – 24.83)	0.020	
**Predicted HRV *index:* ΔHFnu**				0.337
ΔmeanNN	0.373	71.07 (17.05 – 125.08)	0.011	
Propensity score	-0.339	-13.54 (-24.86 – -2.23)	0.020	
**Predicted HRV *index:* ΔLF/HF**				0.251
ΔmeanNN		*Excluded variable*		
Propensity score	0.501	5.09 (2.24 – 7.95)	0.001	
**Predicted HRV *index:* Δα_1**				0.394
ΔmeanNN	-0.445	-1.50 (-2.42 – -0.59)	0.002	
Propensity score	0.321	0.28 (0.35 – 0.42)	0.002	
**Predicted HRV *index:* Δα_{1sign}**				0.228
ΔmeanNN		*Excluded variable*		
Propensity score	0.477	0.243 (0.09 – 0.38)	0.002	

Table S4. Characteristics and risk factors of aortic valve stenosis (AVS) patients. Data are shown as absolute value (percentage), mean ± standard deviation, or median (percentile 25 – percentile 75).

Variable	Moderate AVS (n = 11)	Severe AVS (n = 14)	p value
Age (years)	60.1 ± 6.6	63.4 ± 4.8	0.301
Female<br>Male	3 (27%)<br>8 (73%)	5 (36%)<br>9 (64%)	1.000
Body mass index (kg/m^2)	27.53 ± 2.59	28.97 ± 4.15	0.059
Heart rate (bpm)	62.6 ± 14.6	61.8 ± 8.3	0.198
SBP (mmHg)	137.22 ± 21.02	135.23 ± 20.55	0.592
DBP (mmHg)	80 (76 – 80)	80 (78 – 90)	0.601
Hypertension	7 (28%)	4 (16%)	0.116
Dyslipidemia	4 (16%)	3 (12%)	0.656
Alcoholism	5 (20%)	8 (32%)	0.695
Smoking	4 (16%)	4 (16%)	1.000
Statins	2 (8%)	3 (12%)	1.000
Aspirin	4 (16%)	6 (24%)	1.000

Table S5. Biochemical parameters of the aortic valve stenosis (AVS) patients. Data are shown as mean ± standard deviation or median (percentile 25 – percentile 75).

**Variable**	Moderate AVS (n = 11)	Severe AVS (n = 14)	**p value**
Serum glucose (mg/dL)	102.3 ± 9.53	93.9 ± 11.5	1.000
Albumin (mg/dL)	4.35 ± 0.19	4.50 ± 0.34	0.188
Total cholesterol (mg/dL)	171.05 ± 35.83	190.73 ± 37.50	0.767
High density lipids (mg/dL)	44.42 ± 12.59	41.54 ± 11.23	0.559
Low density lipids (mg /dL)	101.84 ± 31.72	110.41 ± 39.18	0.319
Triglycerides (mg/dL)	158.9 (87.5 – 188.8)	145.6 (105.6 – 234.4)	0.809
Atherogenic index	2.38 ± 0.85	2.98 ± 1.62	0.027
C-reactive protein (mg/dL)	2.0 (0.26 – 9.23)	1.83 (0.89 – 4.17)	0.937
Hemoglobin (mg/dL)	14.42 ± 1.42	14.87 ± 1.57	0.990
Hematocrit (%)	42.46 ± 4.30	44.30 ± 4.80	0.820

Table S6. Heart rate variability indices at supine position and after active standing of the aortic valve stenosis (AVS) patients. Data are shown as mean ± standard deviation, or median (percentile 25 – percentile 75). The groups were compared with analysis of variance for repeated measures or Kruskal-Wallis test, Mann-Whitney U test and Wilcoxon rank's test.

**Variable**	Moderate AVS (n = 11)	Severe AVS (n = 14)	**p value**
Supine position			
MeanNN (s)	1.022 ± 0.18	0.960 ± 0.15	0.634
SDNN (ms)	0.056 ± 0.33	0.050 ± 0.19	0.021
pNN20 (%)	46.30 ± 40.49	37.23 ± 23.17	0.110
RMSSD (ms)	26.91 ± 12.51	25.87 ± 14.36	0.510
LF (n.u.)	54.16 (47.7 – 81.13)	79.66 (74.4 – 87.6)	0.018
HF (n.u.)	45.83 (18.8 – 52.2)	20.33 (12.3 – 25.5)	0.018
LF/HF	1.18 (0.91 – 4.3)	3.93 (2.9 – 7.1)	0.018
α_1	0.96 ± 0.44	1.32 ± 0.34	0.466
α_{1sign}	0.21 ± 0.25	0.42 ± 0.17	0.716
Active standing			
MeanNN (s)	0.90 ± 0.14	0.87 ± 0.11	0.477
SDNN (ms)	0.03 ± 0.01	0.04 ± 0.01	0.974
pNN20 (%)	30.15 ± 26.3	27.9 ± 15.2	0.089
RMSSD (ms)	17.8 ± 12.1	20.1 ± 6.7	0.022
LF (n.u.)	75.8 (65.8 – 85.9)	83.2 (75.1 – 87.3)	0.344
HF (n.u.)	24.1 (14.0 – 34.1)	16.7 (12.6 – 24.8)	0.344
LF/HF	3.1 (1.9 – 6.1)	5.0 (3.0 – 6.9)	0.344
α_1	1.1 ± 0.37	1.3 ± 0.24	0.101
α_{1sign}	0.32 ± 0.15	0.40 ± 0.11	0.203

Table S7. Magnitude (Δ) of change in heart rate variability indices in response to active standing of the aortic valve stenosis (AVS) patients. Data are shown as mean ± standard deviation and were compared between groups by a Student t-test for independent groups.

**Variable**	Moderate AVS (n = 11)	Severe AVS (n = 14)	**p value**
ΔmeanNN (s)	0.112 ± 0.147	0.087 ± 0.072	0.107
ΔSDNN (ms)	0.013 ± 0.026	0.004 ± 0.01	0.247
ΔpNN20 (%)	16.14 ± 29.80	9.24 ± 16.87	0.212
ΔRMSSD (ms)	9.04 ± 11.57	5.81 ± 11.61	0.942
ΔLF (n.u.)	-13.08 ± 21.07	-2.27 ± 8.42	0.083
ΔHF (n.u.)	13.18 ± 21.03	2.24 ± 8.37	0.083
Δ(LF/HF)	-2.08 ± 2.52	-0.035 ± 4.41	0.227
$\Delta\alpha_1$	-0.14 ± 0.38	-0.01 ± 0.24	0.314
$\Delta\alpha_{1sign}$	-0.10 ± 0.22	0.02 ± 0.19	0.737
